# Scoping review on the prioritisation of high-consequence infectious pathogens for research preparedness and response to health emergencies

**DOI:** 10.1186/s12916-026-04789-w

**Published:** 2026-04-01

**Authors:** Nicolas Pulik, Holly Sadler, Thokozani Nyasulu, Richmonda Pearce, Yazdan Yazdanpanah, Hervé Raoul, Alice Norton

**Affiliations:** 1 Policy and Practice Research Group, Pandemic Sciences Institute, Nuffield Department of Medicine, University of Oxford, Oxford, UK; 2 ANRS Emerging Infectious Diseases, Inserm, Paris, France

**Keywords:** Research prioritisation, High-consequence pathogens, Health research systems, Scoping review

## Abstract

**Background:**

Funding for health research is intrinsically limited; therefore, priority-setting is necessary to optimise investments. Prioritisation is particularly important for epidemic and pandemic pathogens, as preparedness needs for hypothetical threats can be difficult to assess, and response during outbreaks requires accelerated decision-making. Exercises which prioritise epidemic and pandemic pathogens have been published; however, to our knowledge, there has been no systematic analysis of how, why and by whom these exercises are conducted. Guidelines and good practices for prioritisation exist, but whether these are followed for pathogen prioritisation and the consequences on the quality and impact of results has not yet been analysed.

**Methods:**

We undertook a scoping review to investigate processes and methods of analysis reported across pathogen priority-setting exercises. We ran a grey literature search on Overton and Google to identify relevant resources published since 2018. The resources were screened by independent reviewers and extracted data were analysed using descriptive statistics and narrative synthesis.

**Results:**

The identified pathogen prioritisation exercises varied in geographical scope and included exercises focusing on specific threats such as antimicrobial resistance, as well as all-hazard prioritisation activities. We identified differences in the types of participants involved and their roles in priority-setting processes. While a diversity of processes and methods of analysis exist, there were common practices and steps, notably identifying candidate priority pathogens and developing assessment criteria, and then scoring the former against the latter to reach a final priority list. There were commonalities in the criteria themes used to score pathogens, such as severity of disease, transmissibility, prevalence, impact and mitigation measures. Rabies and influenza A were the two most reported priority list pathogens. Our ability to synthesise a meaningful ordered priority list across publications was limited by discrepancies in taxonomy and scope.

**Conclusions:**

This study provides the first examination of priority setting exercises identifying high-consequence infectious pathogens. The review reveals important elements of commonality and variability between priority-setting exercises, highlighting areas for improvement to increase the comparability and quality of future efforts and their implementation.

**Supplementary Information:**

The online version contains supplementary material available at 10.1186/s12916-026-04789-w.

## Background

### Introduction

Given that health research funding is limited, research prioritisation is key to efficient resource allocation. Outside of emergency response, research prioritisation is needed to strengthen preparedness by advancing knowledge on potential future threats [1]. During health emergencies, research prioritisation is also key as it addresses urgent and locally relevant questions, which may only be studied during epidemics, such as on vaccine efficacy trials. In some instances, even this emergency research prioritisation can be undertaken in advance to enhance responsiveness to potential epidemics and pandemics. Prioritisation of high-consequence pathogens streamlines investments to ensure key gaps are addressed, and duplication and waste in research are minimised. A recent analysis of research prioritisation relating to high-consequence pathogens has shown that numerous rationales and diverging approaches co-exist such as external consultations, literature reviews and database reviews [1]. While Antonio et al. looked at how research areas, such as therapeutics development or behavioural research, are prioritised for identified pathogens [1], we were interested in the prioritisation of the high-consequence pathogens themselves. These can be defined as “*infectious disease pathogens which cause diseases in humans with the potential to cause outbreaks associated with devastating morbidity and mortality*” [2]. Several lists of priority pathogens have been published in recent years, with discrepancies in the processes and methods used [3–5]. Whilst global pathogen prioritisation exercises have been undertaken by the World Health Organization (WHO) [3], prioritisation at both regional and national levels is also undertaken and important to account for local context. Analyses of research priority setting exercises have been published [6], but we have not identified any assessing how pathogens, rather than research disciplines, are prioritised.

A number of papers have outlined the various methodological [6] or ethical issues [7, 8] that may arise with research prioritisation, such as the heterogeneity in processes, the extent and modalities of stakeholders’ involvement or apparent conflicts between global and local priorities. There is also disparate literature on good practices for priority-setting exercises, including on reporting practices and conceptual frameworks to develop prioritisation exercises [6, 9–11]. Viergever et al. identified nine themes of good practices such as inclusiveness and transparency, as well as an array of existing methods for research prioritisation [6]. A conceptual framework for priority setting has also been proposed to guide prioritisation processes, highlighting ten elements for successful priority setting such as consideration of context and the use of clear processes throughout the exercise [10]. Finally, a set of 31 reporting guidelines to improve transparency of these exercises has also been published, including items such as definition of context, description of the framework used for priority setting and implementation and evaluation practices [11]. The World Health Organization has published guidance outlining good practices for research priority-setting which also identifies various methods for prioritisation and develops a template to guide such processes; it encompasses the important issues mentioned in the literature and proposes and end-to-end process for research prioritisation from planning to evaluation; it is the most comprehensive guide to priority-setting that we have identified [9]. Therefore, while all frameworks or guidelines for priority-setting share common features in terms of reporting and processes, they also put forward various methods to establish priorities in health research, and none is specifically dedicated to the prioritisation of pathogens. Consequently, this review aims to characterise existing priority pathogens lists for infectious diseases research as well as their methods as a first step towards identifying good practice for pathogen prioritisation.

### Rationale

Given the diversity of methods and approaches, we wanted to investigate whether pathogen prioritisation exercises share specific features. Therefore, the objectives of this work were to (i) identify the geographic scope and purpose of existing lists of priority pathogens; (ii) describe the processes, analytical methods and criteria used to develop these lists; and (iii) identify and compare the pathogens prioritised by these exercises.

This work was developed with the aim to inform future pathogen prioritisation approaches, including directly for ANRS Emerging Infectious Diseases (ANRS MIE), a French National Research Funding Agency focusing on infectious diseases which is currently engaged in similar processes.

## Methods

This scoping review was conducted in accordance with the JBI guidelines [12] for scoping reviews and the Arksey and O’Malley framework [13]. The reporting was done according to the PRISMA-ScR guidelines [14].

### Eligibility criteria

The goal of this review is to characterise existing priority pathogens lists for infectious diseases research. Therefore, we included resources whose goal was to establish a list of high-consequence infectious pathogens, amongst which at least one must belong to the WHO Pathogen Prioritisation list (Additional file 1: Table 1) [3]. Prioritisation that focuses on research domains within one specific pathogen were excluded. We only included resources that were published on or after the 1st of January 2018, which year marks the publication of the WHO first list of priority diseases. We did not restrict the type or language of resources we analysed. Additional file 2 summarises the inclusion and exclusion criteria (Additional file 2: Table 2).

### Information sources and search

We searched Overton on the 26th of September 2024 for documents published on or after the 1st of January 2018, using piloted and refined search terms in titles and abstracts (Additional file 3: Table 3). We also searched relevant regional and national public health bodies’ websites through Google Advanced Search tool to complement the initial search (Additional file 3: Table 3). In order to select a list of relevant public health bodies, we used the Pandemic PACT database [15] to identify countries and regions with research investments in high-consequence diseases. For each WHO region, we searched total funding amounts, and the total number of grants supported. We selected the top three funders by funding amounts, or grant numbers when funding amounts were not reported. This enabled us to identify countries that are likely to use priority disease lists for research funding. We then looked for national and regional public health bodies linked to the countries or the region they belonged to (Additional file 4: Table 4). The websites of these institutions were searched to identify priority disease lists, and we screened the first 100 results for inclusion.

### Selection of sources of evidence

The resources were identified through two rounds of screening — first title and abstract and then full-text using Rayyan [16] and Microsoft Excel [17]. The resources were reviewed for eligibility according to the inclusion and exclusion criteria. Conflicts were resolved by a third reviewer.

### Data charting process and data items

The data was extracted using Microsoft Excel [17]. The data extraction form was piloted, reviewed and pre-agreed upon with the research team. We extracted general information (title, authors, date), the methods used for prioritisation, the results of the priority setting exercise, and aspects related to the monitoring and evaluation framework from the WHO guidance for research priority setting [9]. Additional file 5 describes in detail the data extraction framework used (Additional file 5: Table 5), and the data extraction sheet was published [18].

### Synthesis of the result

The results were analysed and presented using descriptive statistics and framework analysis.

#### Stakeholder involvement in pathogen prioritisation

We defined a stakeholder as ‘a person that gives a direct personal input into the prioritisation process and has an impact on the final output’; this could be at various stages of the process. Facilitators, technical supporters and organisers were not included in the count, given their neutral role, unless they were explicitly mentioned to have contributed to the prioritisation process.

#### Process and methods of pathogen prioritisation

We extracted the processes and methods used throughout the resources and classified them according to steps emerging from the resources. We used a process chart to represent discrepancies and similarities.

#### Criteria themes and weighting for priority pathogens

The criteria the resources used to assess priority pathogens were grouped under mutually exclusive emergent themes. Criteria which addressed multiple themes were considered under all relevant themes. For each resource which weighted criteria as part of the prioritisation process, the relative weights assigned to each of our criteria themes was calculated to identify which themes were considered to be of greatest importance. If multiple criteria were used to assess a theme, the weights were summed. If a criterion addressed multiple themes, the weight for that criterion was split across the relevant themes in proportion to their contribution to the criterion scoring system. For instance, if half of the points for the criterion score related to theme 1 and half to theme 2, the weight of the criterion was split evenly amongst them.

#### Priority pathogens identification

The pathogens in the final prioritised list from each resource were extracted. For one resource [5], which ranked all the pathogens under consideration, this therefore included all pathogens considered. For the others [3, 19–29], the final list included only a subset of the pathogens considered. The pathogens listed were consolidated according to how they were most often reported, typically at the genus level for bacteria and fungi, with some exceptions when we considered that the species were of public health relevance.

Where groups of pathogens were listed without naming specific pathogens, the group was recorded as reported in the resource. Where groups were listed with specific members named, we extracted the individually named pathogens. For instance, for one resource which listed “*SARS-CoV, SARS-CoV-2, MERS-CoV, and other highly pathogenic human coronaviruses*”, we extracted each of the three named pathogens and a separate instance for “*other highly pathogenic human coronaviruses*”. Pathogens were then grouped into broad categories according to phylogeny, disease type, or to align with groupings used in the resources. The visualisation was created with Flourish [30]. As resources included a mix of ranked and unranked lists, methodological challenges prevented us from meaningfully identifying top ranked pathogens.

#### Alignment to existing best practice

We assessed the quality of the identified resources by mapping them against the WHO systematic approach for undertaking a research priority-setting exercise, which constitutes the most encompassing and comprehensive framework we have encountered [9]. Some questions were adapted to match the context. The extraction framework can be found in Additional file 6 (Additional file 6: Fig. S6).

## Results

### Selection of sources of evidence

The Overton search returned 1072 results. After the first round of screening, 1058 resources were excluded (98.7%) and 14 were included (1.3%). The public health bodies websites search returned 849 results. After the title and abstract screening, 841 resources were excluded (99.1%) and eight resources were included (0.9%). Most resources were excluded because they lacked the pathogen prioritisation aspect.

This gave a total of 22 resources sought for retrieval. One could not be retrieved because the webpage had become inactive. During full-text screening, the remaining 21 resources were assessed for eligibility; six were excluded because they had no priority lists and three were duplicates. The WHO prioritisation exercise [3] was added from previous identification as it motivated this review. It was likely not captured by the Overton search due to its recent publication and was out of the scope of the Google search. The flowchart summarising the selection of the 13 sources can be found in Additional file 7 (Additional file 7: Fig. S7). The results of individual sources of evidence and characteristics of these sources have been published online [18].

### Synthesis of results

#### Presentation of the sources of evidence

The resources included in this study were all published between 2018 and 2024, with four being published before the start of the COVID-19 pandemic [26–29] and 9 published after [3, 5, 19–25]. Seven resources were linked through a common methodology developed by the United States Centers for Disease Control and Prevention (US CDC) to prioritise zoonotic diseases [31], which has been applied by countries in collaboration with US CDC [21, 23, 24, 26–29]. The remaining six resources described independent exercises with their own methodology [3, 5, 19, 20, 22, 25]. Two exercises were conducted by an intergovernmental organisation (WHO) and generated global priority lists [3, 20] — one for epidemic and pandemic preparedness, and the other for antimicrobial resistance research (AMR). One exercise was conducted by a regional authority (Africa Centres for Disease Control and Prevention) and identified regional priorities for epidemic-prone diseases for emergency preparedness and response [5].

**Fig. 1:**
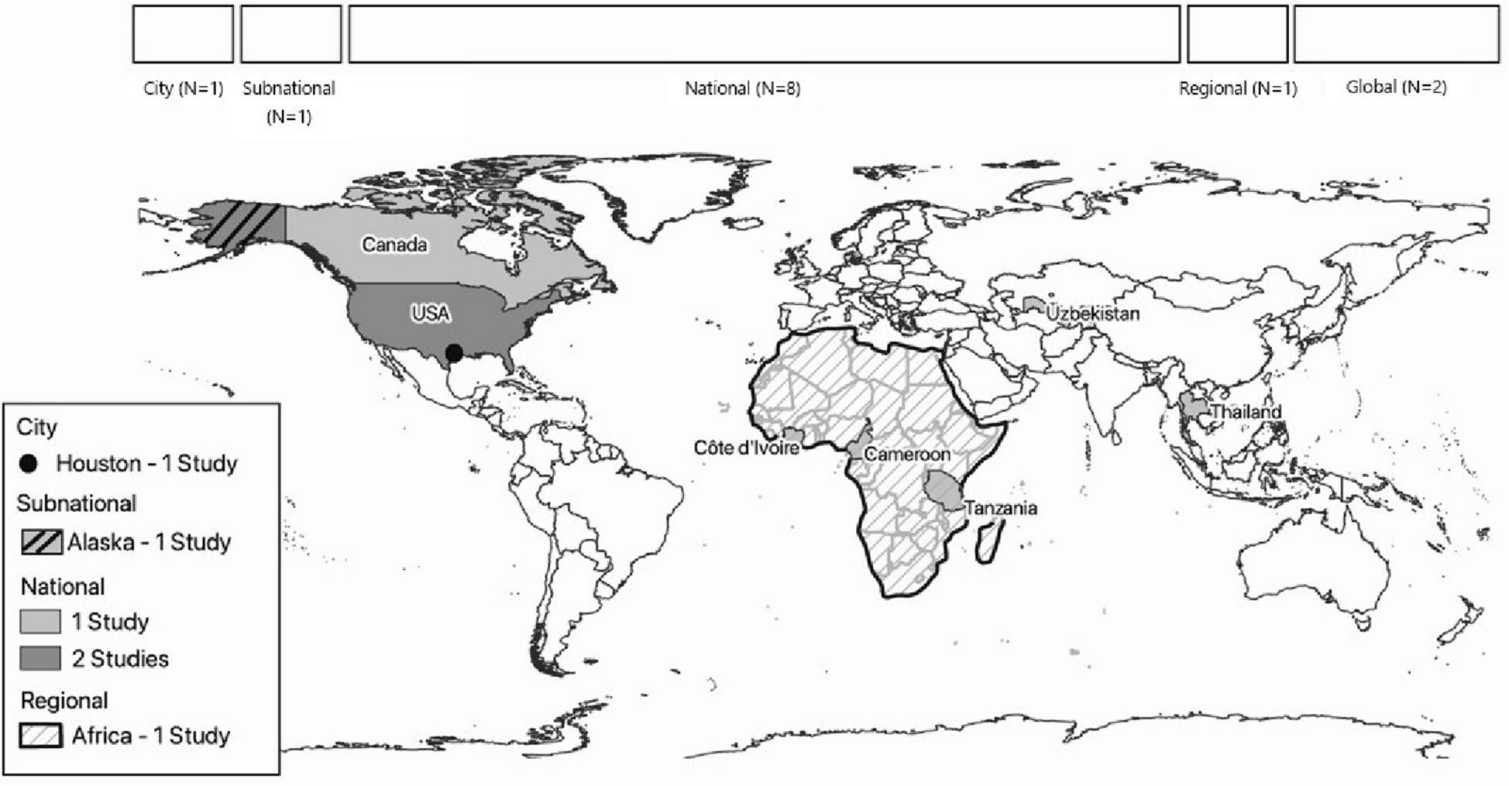
Map representing the geographical scope covered by the included resources

The majority of resources reflected national prioritisation [19, 21, 23, 25–29], including six of the seven US CDC-linked exercises on zoonotic diseases, which were conducted in Cameroon, Côte d’Ivoire, Uzbekistan, Tanzania, Thailand and the United States (US). In addition, Health Canada published a priority list for AMR therapeutics development and the US National Institute for Allergy and Infectious Diseases (NIAID) published a biodefence list. Two exercises addressed subnational priorities within the US [22, 24] — one in which the US CDC framework was applied to the state of Alaska, and another which prioritised pathogens for wastewater surveillance in the city of Houston, Texas (Fig. 1).

#### Stakeholder involvement in pathogen prioritisation

**Fig. 2:**
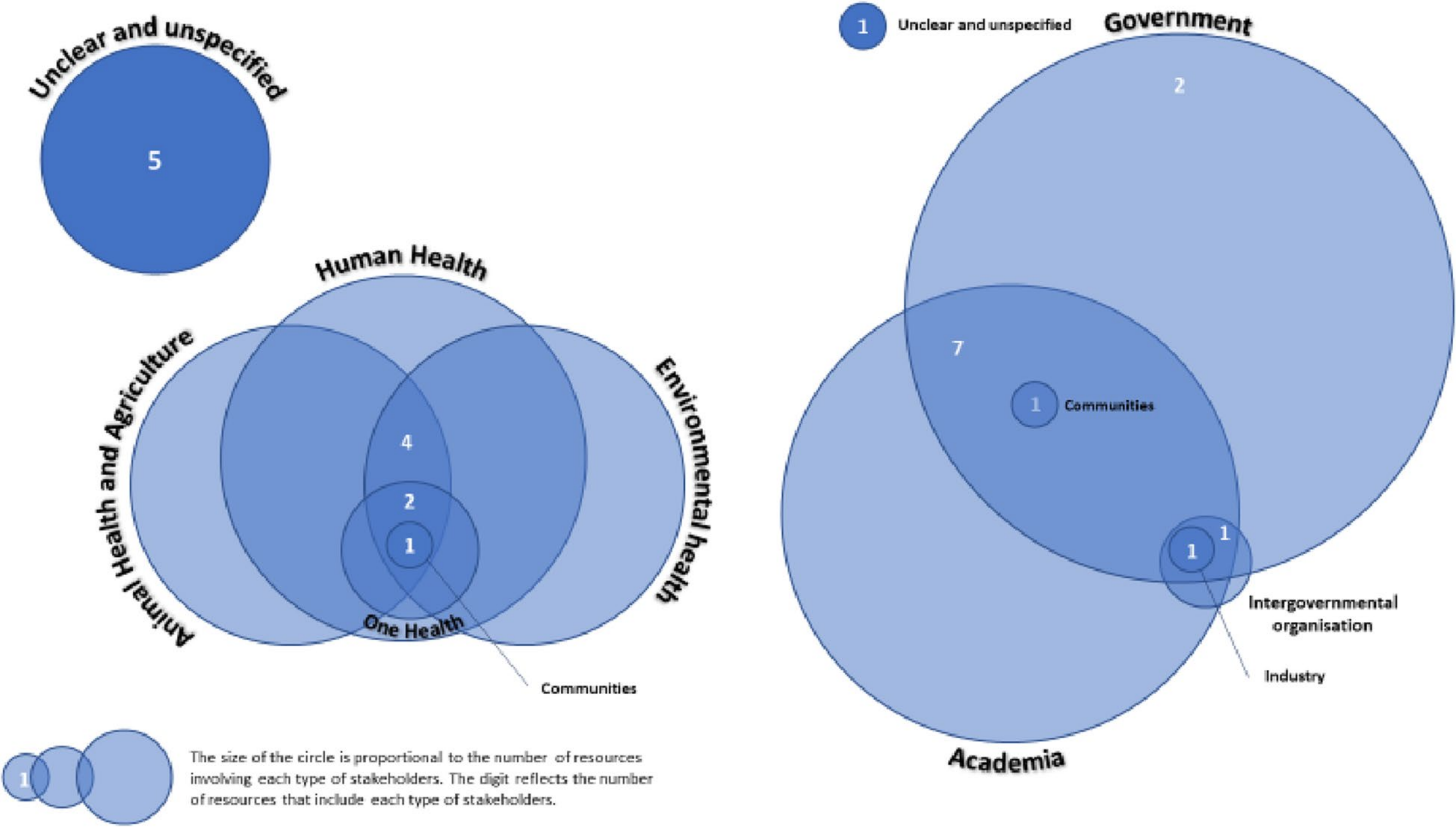
Diagrams representing the stakeholders involved in the prioritisation process depending on their affiliation and expertise

All thirteen resources reported on stakeholders’ involvement [3, 5, 19–29]. We found that stakeholders were characterised in two different ways: their expertise/domain (e.g. animal health, human health) and/or their affiliation (e.g. academia, government) (Fig. 2). Five resources did not state the expertise of the stakeholders involved [3, 5, 19, 20, 25]. The resources that did report on expertise tended to have multidisciplinary stakeholders, with seven [21, 23, 24, 26–29] of the eight involving human health, animal health and agriculture, and environmental health experts.

The affiliation of participants was documented in twelve resources [3, 5, 20–29], with ten exercises involving participants from both government and academia [3, 5, 20–22, 24–28]. Of these ten, three involved stakeholders from one or two additional sectors [3, 20, 24]. Two resources involved only participants from the government [23, 29]. International organisations were represented in two resources [3, 20]. Community involvement was reported in one resource [24], and industry involvement in another [3].

Eleven resources reported on the number of stakeholders involved in their prioritisation process [3, 5, 20–24, 26–29] and this ranged from 17 [27] to 206 [3]. The number of participants also varied within resources, with different groups being assigned distinct tasks along the process in most (*n* = 10/13, 77%) [3, 5, 20, 21, 23, 24, 26–29]. For instance, the US CDC resources (*n* = 7/13, 54%) had a small voting group composed of nine to 12 stakeholders, which was used to weigh the criteria [23, 24, 26–29], agree on the refined list of diseases (*n* = 2/7, 29%) [21, 29] or validate the final list of priorities (*n* = 4/7, 57%) [26–29]. Other steps included additional or alternative voting participants, or additional observers. There were also instances of two independent groups being involved in the process, such as in the WHO Bacterial Prioritisation exercise where a survey was distributed to 79 experts to determine criteria weights while the rest of the exercise was undertaken by separate advisory group in coordination with WHO staff [20]. Another example is the WHO Scientific Framework for Epidemic and Pandemic Research Preparedness which involved Pathogen Family Experts Groups in the first step of the exercise and a Prioritisation Advisory Committee to review and finalise the result [3]. This demonstrates the heterogeneous approaches to inclusion of stakeholders between and within resources. There did not seem to be a link between the stakeholders’ domain and the task attributed to them; however, in five resources, only government representatives were involved in voting [21, 23, 24, 26, 29] and overall four resources did not report on whom the voting members were [5, 19, 20, 25].

#### Process and methods of pathogen prioritisation

**Fig. 3:**
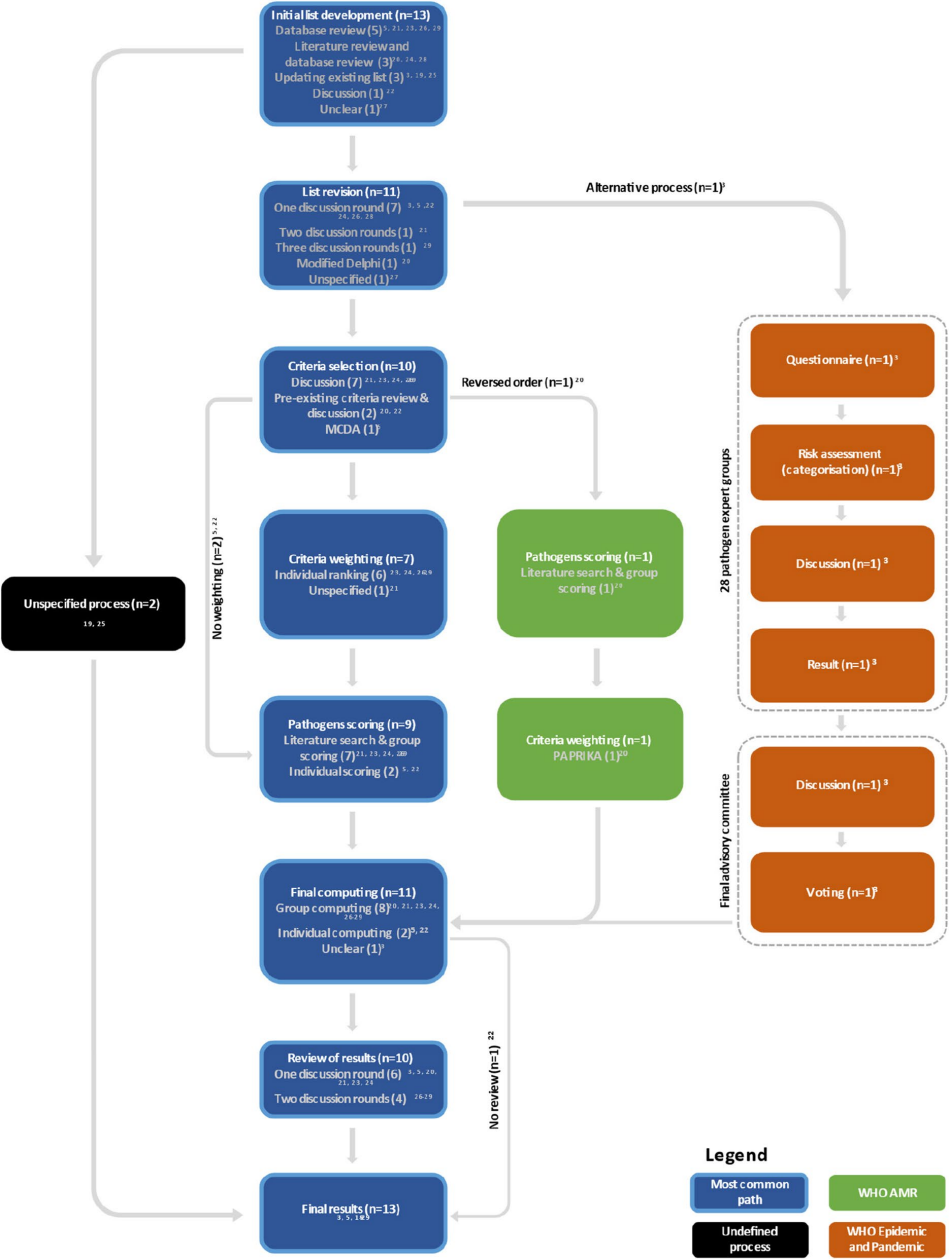
Process chart representing differences in prioritisation processes

We used a process chart to represent the various processes and methods of analysis used across resources (Fig. 3). While most resources used similar steps, the order of these and the methods used at each step varied. All 13 resources started by developing an initial list of pathogens to be considered for prioritisation [3, 5, 19–29]. Five resources used database review [5, 21, 23, 26, 29], three used both database review and literature review [20, 24, 28], three updated an existing list of pathogens [3, 19, 25], one used a discussion [22], and one was unclear on the way this was achieved [27]. From this common starting point, two resources did not specify their process and gave only a final result [19, 25]. In the other cases, the pathogen list was revised before proceeding to full assessment (*n* = 11/13, 85%) [3, 5, 20–24, 26–29]. This step was in most cases organised around stakeholder discussions (*n* = 9/11, 82%) [3, 5, 21–24, 26, 28, 29].

**Fig. 4:**
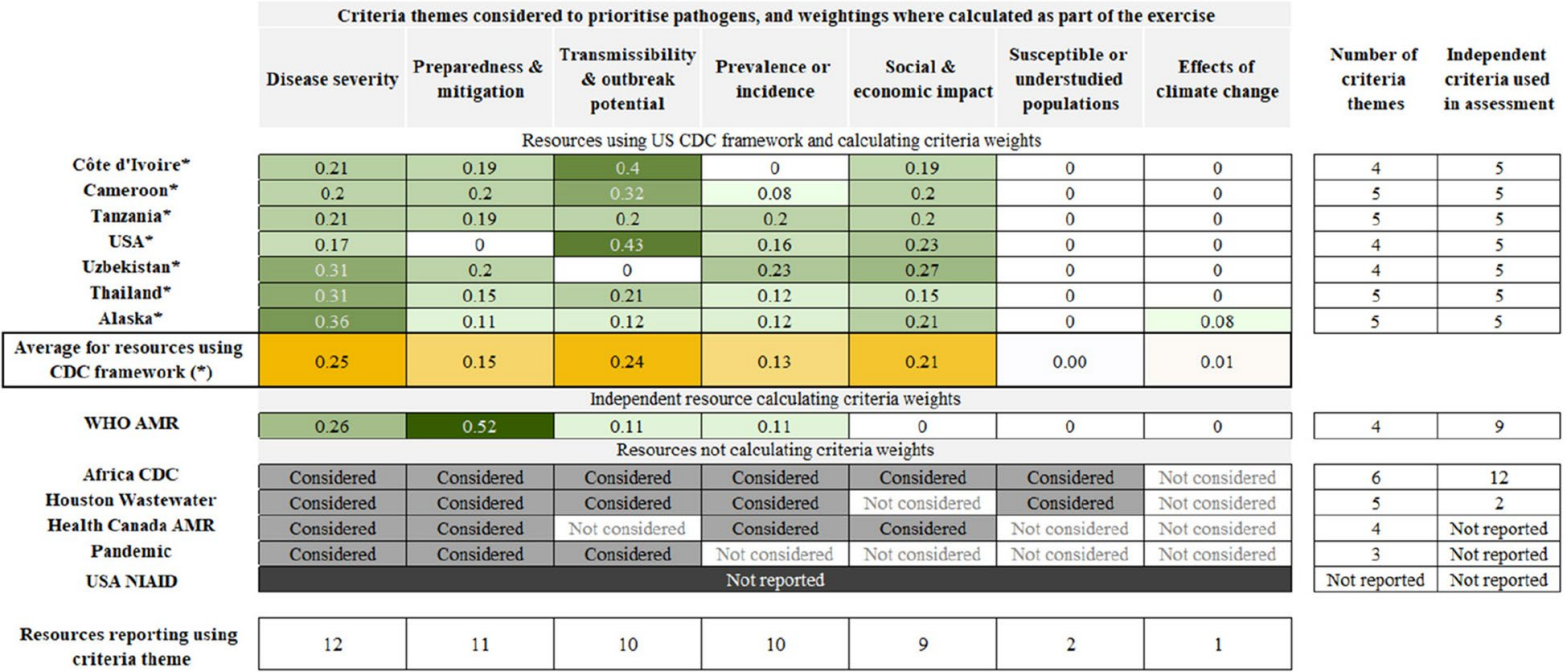
Criteria themes considered to prioritise pathogens, and weightings where calculated as part of the exercise

Once the initial list of pathogens was agreed upon, most resources moved to criteria selection (*n* = 10/11, 91%) [5, 20–24, 26–29]. The WHO Scientific Framework for Epidemic and Pandemic Research Preparedness followed a different process: a first pathogen family step to identify relevant pathogens and then a second higher-level review of these lists, detailed in Fig. 4 [3]. Most resources used discussion to decide on assessment criteria (*n* = 7/10, 70%) [21, 23, 24, 26–29]. Two resources reviewed already existing criteria and discussed these [20, 22] and one resource used multiple-criteria decision analysis [5]. The next step was typically the weighting of the criteria (*n* = 7/10, 70%) [21, 23, 24, 26–29], though two resources did not apply weighting, implicitly giving all criteria the same weight [5, 22], and one resource started by scoring the pathogen and then weighted the criteria later [20]. The criteria weighting was mainly done through individual ranking of criteria (*n* = 6/8, 75%) [23, 24, 26–29], except one resource which used the *Potentially All Pairwise RanKings of all possible Alternatives* method (PAPRIKA) [20] and one which did not specify a process [21]. Then, exercises moved to pathogen scoring (*n* = 10/10, 100%) [5, 20–24, 26–29], which was done using a literature search and group scoring in most cases (*n* = 8/10, 80%) [20, 21, 23, 24, 26–29], only two resources had stakeholders score individually [5, 22].

For the final computing of the results (*n* = 11/13, 85%) [3, 5, 20–24, 26–29], the majority of the resources used group computing by which they multiplied the score the pathogen was given by the group on a specific criterion by the weight given to this criterion, repeated this for all criteria and summed, and then normalised to one (*n* = 8/11, 73%) [20, 21, 23, 24, 26–29]. Two resources used individual computing; one used the sum of scores and also the average of scores across participants to compute the final list [22], the other cross-multiplied the averages of all participant scores for two criteria to get a final score [5]. One resource did not describe how results were computed [3].

After the results were calculated, almost all resources finalised their priority lists using a qualitative discussion step (*n* = 10/11, 91%) [3, 5, 20, 21, 23, 24, 26–29], which included one [3, 5, 20, 21, 23, 24] or two rounds of discussion with different groups of stakeholders [26–29].

#### Criteria for prioritising pathogens

Ten resources clearly reported the criteria which were independently scored to assess pathogens; the seven US CDC-facilitated resources each used five criteria [21, 23, 24, 26–29], while the other three resources used two [22], nine [20] and twelve [5] criteria, respectively. Two other resources reported the considerations broadly used for assessment, but did not report how they were used [3, 19]. One resource did not provide information on the criteria used [25].

We have classified the criteria used into five major themes, which we defined as: disease severity, prevalence or incidence, transmissibility and outbreak potential, preparedness and mitigation, social and economic impact; and two minor themes: susceptible and understudied populations, and the effects of climate change (Fig. 4). Where described, resources addressed between three and six of these themes. However, some criteria addressed multiple themes, and the same theme was sometimes addressed through multiple criteria, so that there is not a one-to-one relationship between the number of criteria used in a resource and the number of themes addressed.

*Disease severity*, ‘the extent to which the pathogen causes serious disease in animals or humans’, was considered in all resources where criteria were reported (*n* = 12/13, 92%) [3, 19, 21, 23, 24, 26–29]. Examples of criteria used to assess this theme included the infection fatality rate, the proportion of severe cases, lived years with disability for non-fatal outcomes, and pathogen virulence.

*Preparedness and mitigation*, ‘the extent to which the threat can be managed or mitigated, including the strength of current knowledge’, was considered in eleven resources [5, 19–21, 26–28]. Criteria referred to the availability of vaccines, therapeutics, effective public health and social measures, diagnostics, and animal models and reagents for research. Development pipelines were also considered. Lack of knowledge on pathogen characteristics and trends in antimicrobial resistance were considered as potential threats to preparedness efforts.

*Transmissibility and outbreak potential*, ‘the extent to which the pathogen has the potential to spread and the perceived risk of causing outbreaks’, was assessed in ten resources [3, 5, 20, 22–24, 26–29]. Examples included whether the pathogen has caused an outbreak in the country or region in the last 10 years, could lead to a cross-country outbreak, and the mode of transmission between animals and humans.

*Prevalence and incidence*, ‘the extent to which the pathogen is present in the area of interest’, was assessed in ten resources [5, 19–24, 27–29]. Criteria included both known and predicted circulation patterns, such as whether the disease has been reported in the country or region in the last 10 years, whether the pathogen has endemic or epidemic status, the global incidence of cases, and the perceived probability of the pathogen circulating among humans in the next 5 years.

*Social and economic impact*, ‘the extent to which a pathogen has the potential to cause social, economic or wider impacts’, was addressed in nine of the resources [5, 19, 21, 23, 24, 26–29]. Criteria were sometimes broadly defined in terms of *“social impact*”, the “*economic impact of a 1000 cases outbreak*” or the “*impact on animal production and trade*” and sometimes were more specific, for instance, whether the pathogen results in quarantine or is on a bioterrorism threat list.

*Susceptible or understudied populations*, ‘the extent to which some parts of the population may be expected to experience particularly poor outcomes or in whom the current risk is not well characterised’, was assessed in only two resources [5, 22]. One resource considered how many regions in Africa have large pools of highly susceptible populations for this pathogen [5]. The other resource, which prioritised pathogens for enhanced wastewater surveillance, considered vulnerable populations underrepresented in other surveillance approaches, which could indicate a stronger public health need for surveillance through wastewater [22].

The expected effects of *climate change* and other environmental drivers on the disease over the next 10 years were considered in one resource [24].

#### Weighting of criteria

Eight resources assigned relative weights to criteria as part of the pathogen prioritisation process [20, 21, 23, 24, 26–29]. Across the seven US CDC-facilitated resources assessing zoonotic risks, disease severity and transmissibility/outbreak potential were the two criteria themes most often given the highest weighting [21, 23, 24, 26–29]. The WHO exercise which prioritised bacterial pathogens with an antimicrobial resistance focus gave more weight to current and future preparedness and included four independently assessed criteria under this theme: treatability in the community, medicines and diagnostics pipeline, preventability in the community, and trend of resistance [20]. Disease severity was the second-highest weighted criteria theme in this exercise [20]. This is summarised in Fig. 4.

#### Priority pathogens identified

**Fig. 5:**
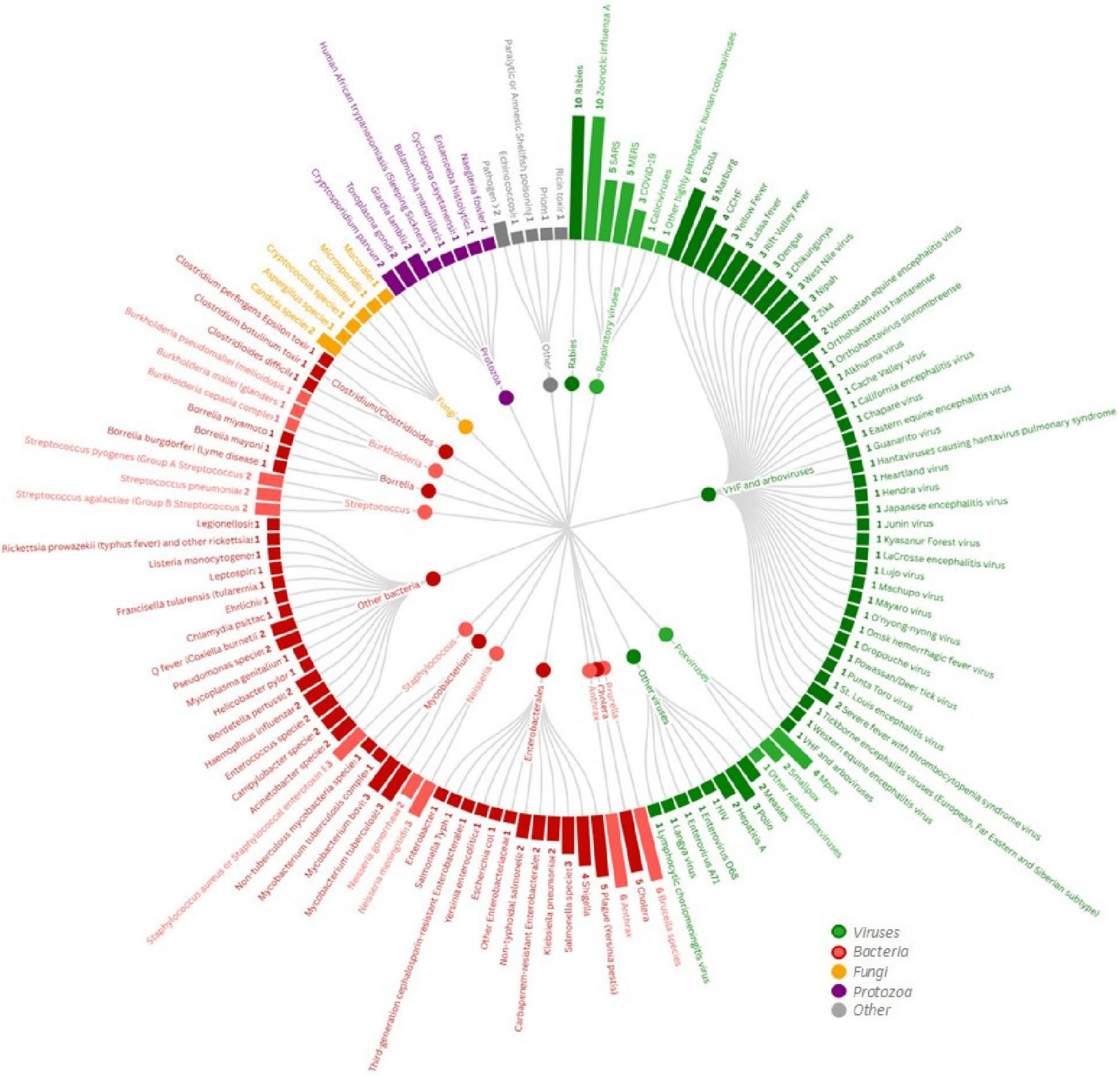
Prioritised pathogens, diseases or other agents, by number of appearances in priority pathogen lists. Note: Pathogens are listed at different taxonomic levels across studies, including phenotype, species, genus and order, as well as disease and broader classifications covering multiple groups (such as “viral hemorrhagic fevers and arboviruses”). Therefore, the number of appearances for each pathogen does not sum to the total number of pathogens listed across all studies. As far as possible, items are listed as the causative pathogen and at the same taxonomic level as they are most often listed in the resources

The list of priorities identified vary greatly in length, taxonomy and scope. The number of items prioritised within one list ranged from five [23, 26–28] to 85 [25], with an average of 18 items (noting these ‘items’ were at variable taxonomic levels). Figure 5 shows the identified pathogens (or occasionally diseases or other agents such as toxins where a causative pathogen could not be directly extracted) grouped into broad categories according to phylogeny, disease type or to align with groupings used in the resources.

Rabies [5, 21–29] and influenza [3, 21–29] were the most often listed viruses and pathogens overall, appearing in 10 resources each. 38 different viral haemorrhagic fevers (VHFs) and arboviruses were featured across 10 resources [3, 5, 21–23, 25–29], with Ebola and Marburg viruses being most often named as priority pathogens with six [3, 5, 23, 25, 27, 28] and five [3, 5, 25, 27, 28] mentions respectively. One resource listed VHFs and arboviruses generally as a priority [26]. Exercises which considered emerging coronaviruses often prioritised them as a group; however, SARS (*n* = 5/13, 39%) and MERS (*n* = 5/13, 39%) were listed more often than SARS-CoV-2 (*n* = 3/13, 23%), noting that two of the three resources which prioritised MERS and SARS but not SARS-CoV-2 were published prior to 2019. Poxviruses including Mpox (*n* = 4/13, 31%) [9, 11, 22, 25] and smallpox (*n* = 2/13, 15%) [9, 25] also appeared regularly.

*Mycobacterium* species appeared eight times across 6 resources, with three resources specifically referring to *Mycobacterium tuberculosis* [20, 25, 26] or human tuberculosis, three to *Mycobacterium bovis* [21, 26, 27] or bovine or zoonotic tuberculosis, one referring to *Mycobacterium tuberculosis* complex which includes both these species [19], and one referring to non-tuberculous species [19].

Members of the bacterial order Enterobacterales appeared in several resources (*n* = 8/13, 62%). *Yersinia pestis* (plague) was prioritised in five resources [3, 5, 21, 25, 29], *Salmonella* species in five [3, 19, 20, 25, 29], *Shigella* in four [3, 19, 20, 25], and two exercises prioritised Enterobacterales as a larger group [20, 22] according to antimicrobial resistance profile in addition to naming specific pathogens.

The other most commonly prioritised bacterial pathogens were *Brucella* species (*n* = 6/13, 46%) [21, 24–26, 28, 29], Anthrax (*n* = 6/13, 46%) [5, 21, 22, 25, 27, 28], and Cholera (*n* = 5/13, 38%) [3, 5, 19, 22, 25].

Protozoa and fungi made relatively few appearances; *Cryptosporidium parvum* [24, 25], *Toxoplasma gondii* [24, 25] and *Giardia lamblia* [24, 25] appeared twice each, and *Candida* was the only fungal pathogen to appear more than once (*n* = 2/13, 15%) [19, 22].

#### Alignment to existing best practice

**Fig. 6:**
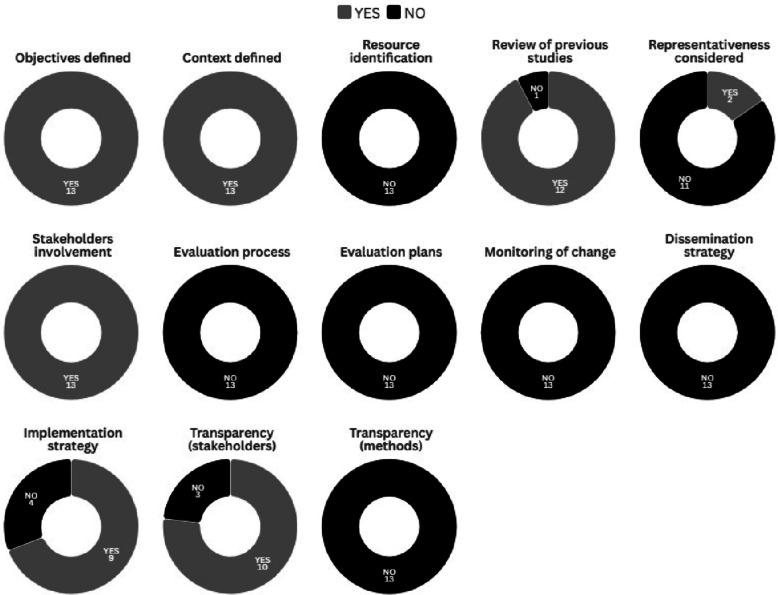
Alignment to the WHO guidelines for undertaking priority setting exercises

Finally, we were interested in understanding if the priority-setting exercises followed established good practices and guidelines. The WHO guidance for a systematic approach to priority-setting exercises details a systematic framework which we used to assess key elements from the resources [8]. Our analysis of alignment of the identified priority-setting exercised to the WHO guidance shows that while some good practices were always observed (objectives and context definition, involvement of stakeholders), others were never followed (human and financial resources identification for the exercise, evaluation plan and process, monitoring of impact and change, dissemination). While internal documents may take these elements into account, they are not reported publicly. Regarding transparency, while stakeholders were described in ten resources, the methods were all unclear and not reproducible, therefore we have split the category in two to reflect this. We have added an implementation category, since all US CDC supported documents included an implementation consideration in the original methods (*n* = 9/13) [31]. Figure 6 summarises the findings.

## Discussion

### Insights from findings

In the context of limited availability of funds for global health research and the necessity to effectively prioritise pathogens for the competitive allocation of research grants, this scoping review characterises the processes, methods and results of existing pathogen prioritisation exercises.

Firstly, this review identifies the heterogeneity in the geographical level at which prioritisation exercises are undertaken. While the majority happen at the national level, some lists have a regional or a global scope. This informs the implementation, acceptability and relevance of these priorities at various geographical levels. This variation in scope also has consequences on the final prioritisation lists, as some consider local risks and vulnerabilities as criteria for pathogen selection. Overall, these limit the transferability of lists between countries or geographic levels; however, shared frameworks such as the US CDC methodology for zoonotic pathogens can be adapted to reflect local perspectives using locally developed criteria. Notably, the WHO priority list includes regional lists in addition to a global list of pathogens, in recognition of regional variation in circulation patterns [3], and while the Africa CDC all-hazards exercise was developed to inform its own strategic planning, it recognises the value of its tool as a shared evidence-based framework for aligning prioritisation activities conducted by national authorities and other partners [5].

Regarding the stakeholders involved in prioritisation processes, the resources include a variety of actors from diverse disciplines and various affiliations. The number of actors and their role in the process vary between resources, even though there seems to be consistency in the involvement of governments and academia across relevant disciplines. Further questions remain in terms of stakeholders’ responsibilities in the different steps of the process and potential issues around the independence of stakeholders. For instance, if participants both weight and score the criteria, this could create incentives to score pathogens according to the weights that were given to the criteria, or give more weight to some criteria because of their own field of expertise, and this would influence the final results. Another consideration around stakeholders is that none of the resources mentioned including policy-makers or research funders, a finding corroborated in another study looking at research priority-setting in health in low- and middle-income countries [32]. Doing so might have a positive impact on implementation and funding practices. Another question relates to the use of evidence-based elements to score pathogens objectively as opposed to using expert opinion; it is unclear which is the most appropriate or how they should be optimally combined, and practice displays variability.

Although prioritisation exercises display similar processes and steps, the methods of analysis used to prioritise pathogens differ between the resources. The criteria used in these exercises tend to share common themes such as severity of disease, transmissibility, prevalence, impact, and mitigation measures. However, the weight of these criteria varies across resources, which could indicate differing perceptions across countries and fields of expertise, or which might also hint at the difficulty to weight criteria resulting in varying weights both between experts participating in an individual exercise and across prioritisation exercises. This also highlights those lists of priority pathogens are highly contextual and might need to be updated as new pathogens emerge, climate factors change or demographic shifts happen. Another area of interest would be to look into the usefulness and impact of these priority lists in the long-term. We suggest further research could be undertaken to understand how these priority lists are implemented, monitored and evaluated, including the impact on funding flows for specific pathogens. We found that only the US CDC exercises were undertaken within a One Health approach involving stakeholders from human, animal and environmental health, despite the potential relevance of One Health to other exercises, for instance those considering the threat of antimicrobial resistance. Furthermore, climate change was only taken into as a criterion in one study and given the current trends this might need to change in future priority-setting exercises. Finally, the questions and vocabulary used to determine criteria weight vary substantially, and this might lead to differences in appreciation and results, beyond the use of different criteria.

Given the variety of analytical methods used and the various groupings used to report the final lists of pathogens, it is difficult to synthesise a meaningful list and ranking of these pathogens. Nonetheless, our analysis showed that some pathogens such as rabies, zoonotic influenza A and groups of pathogens such as viral haemorrhagic fevers tend to be considered consistently across resources. This means that even though the lists differ, they share some commonalities in the pathogens prioritised. This is notably true when focussing on a single region; the four resources generated in African countries share strong similarities [5, 26–28]. This might hint at the fact that the regional level could be scientifically relevant for pathogen priority setting; however, national level engagement is needed for implementation through legal provisions. For example, rabies ranks highly due to several factors: in low- and middle-income countries (LMICs), there is a well-recognised lack of diagnostic capacity, and although pre- and post-exposure prophylaxis is available, it remains costly. Additionally, rabies is associated with severe outcomes once symptoms appear and has a high prevalence in LMICs. In contrast, in high-income countries (HICs), rabies tends to receive lower scores than influenza, which, despite its lower clinical severity when compared to rabies, is recognised for its significant epidemic potential and substantial social and economic impact [33].

Furthermore, it appears that guidelines for undertaking priority-setting exercises are not followed consistently. While some good practices such as defining the context and objectives or reporting stakeholder involvement appear to be followed consistently, others are less often considered such as transparency and reproducibility. This has been highlighted in other reviews looking into research area prioritisation [1]. Most reviews go through similar steps: identification of list of pathogens, definition of contextually relevant criteria, ranking and weighting these criteria, scoring the pathogens against the criteria, discussing and providing a final list. The final discussion step tends to override the previous quantitative stage and brings substantial modifications to the list of pathogens being prioritised, as exemplified in the collaborative US CDC resources. One major unknown is how these discussions are structured and how they complement the quantitative work, so that the effort is not duplicative and contradictory. This review identifies avenues for potential improvements.

Finally, two resources mention pathogen X or an unknown agent [3, 5] and two mention emerging viruses [23, 29], recognising that some priorities might be unknown to the world and that some threats cannot yet be characterised but still need to be accounted for in preparedness planning. This highlights that while most priority lists try to unveil uncertainty in the long-term, they also recognise the need for flexibility and adaptation to new threats. It raises the question of what mechanisms should be used to update lists, and of the time horizon for review. For instance, the WHO bacterial resource reflects on the suitability of the methods for running frequent updates [20], and the Health Canada and NIAID resources mention they will run reviews [19, 25]. The WHO framework focuses on a pathogen family approach, identifying model pathogens for research with applicability to related but yet unknown emerging threats [3]. This prototype pathogen approach is useful for cross-pathogen research for epidemic and pandemic preparedness as it allows repurposing and transfer of knowledge from pathogens that share common characteristics, hence providing a head-start in rapid response; however, pathogen specific research is also key for areas such as viral pathogenesis and natural history of the disease for instance. For the ANRS MIE, this study has proved extremely useful to inform their prioritisation of high-consequence infectious pathogens. Important insights of relevance to similar agencies include the consideration of the merits of the various methods and the need to refer to existing guidelines and good practices throughout the priority-setting exercise.

### Strengths and limitation

This scoping review is, to our knowledge, the first to investigate prioritisation approaches for infectious pathogens. This review proved to be a useful exercise to review methods and processes used for prioritisation. A strength of this study is the combination of two literature searches to capture relevant grey literature.

This study also has limitations. Some resources published in collaboration between the US CDC and national partners have not been captured by our searches. One possible reason for this might be that some were not published on Overton at the time the search was undertaken. Another limitation is that, while the Overton search has identified resources from Latin America and South-East Asia, none met the illegibility criteria as they did not contain lists of priority pathogens, so these areas are not represented in our analysis. We are aware that some lists exist, including a regional exercise for South-East Asia [34]; and the fact that this was not captured by the search is likely to be due to the fact that Overton do not scrap the Association of South-East Asian Nations website. The complementary search using Google Advanced Search was focused on the biggest funders by WHO region, which is why it did not capture funders from Latin America; the lack of transparency on funding sources might have also impacted the search. This appears to suggest that while some lists of priority pathogens exist, they might not always be published or disseminated, perhaps being kept unpublished for specific reasons (for instance lists of priority pathogens with a national security purpose). Furthermore, the great diversity in approaches and variations in length, scope, ranking system and taxonomy of final priority lists make it difficult to do a metanalysis of the priority pathogens themselves.

## Conclusions

In conclusion, this scoping review highlights the variability in approach, quality and transparency in the vital practice of pathogen prioritisation. While pathogen prioritisation activities typically follow similar processes and consider similar criteria themes, such as severity, mitigation measures, prevalence, transmissibility or impact; there are differences in assessment methodologies and in the taxonomic level at which pathogens are considered. This prevents priority pathogens from being effectively compared across time and space; however, there still appears to be an overall agreement on some priority pathogens such as rabies, influenza A and several viral haemorrhagic fevers and arboviruses. There is no universal answer as to which methods should be used for this type of prioritisation exercise. However, maximising the impact of these exercises likely requires better involvement from varied and relevant stakeholders as well as clarity and distinction in their purpose. There is a clear lack of effective guidelines for reporting and standardising processes which have the potential to increase comparability of pathogen prioritisation exercises; it is paramount to bridge that gap. Finally, evaluating and assessing the effectiveness of prioritisation processes is an area yet to be studied.

## Supplementary Information

Additional file 1: Title: List of priority pathogens published by the World Health Organization in 2024. Description: Table of priority pathogens by WHO.

Additional file 2: Title: Inclusion and exclusion criteria. Description: Table of inclusion and exclusion criteria.

Additional file 3: Title: Search strategies for Overton and Google Advanced Search Tool. Description: Table with the search strategies used for the study.

Additional file 4: Title: List of national and regional public health bodies. Description: Table with the national and regional public health bodies that were searched.

Additional file 5: Title: Data extraction framework. Description: Table representing the data extraction framework used for this study.

Additional file 6: Title: Alignment to best practices framework. Description: WHO framework used for analysing the best practices.

Additional file 7: Title: PRISMA-ScR Flowchart. Description: Flowchart describing the process of inclusion and exclusion for this study.

## Data Availability

The datasets generated and/or analysed during the current study are available in the Figshare repository: - Data extraction: [10.6084/m9.figshare.29582636.v1] All data generated or analysed during this study are included in this published article and its supplementary information files.

## Abbreviations

WHO: World Health Organization
ANRS MIE: ANRS Emerging Infectious Diseases
AMR: Antimicrobial resistance
US CDC: United States Centers for Disease Control and Prevention
US: United States
NIAID: National Institute of Allergy and Infectious Diseases
PAPRIKA: Potentially All Pairwise RanKings of all possible Alternatives
Africa CDC: Africa Centres for Disease Control and Prevention
LMICs: Low- and middle-income countries
HICs: High-income countries
