# Supplementary material for: Scoping review on the prioritisation of high-consequence infectious pathogens for research preparedness and response to health emergencies
Source: BMC Med. 2026 Apr 1;24:301. doi: 10.1186/s12916-026-04789-w (PMC13169742; doi:10.1186/s12916-026-04789-w)
Supplement: Supplementary file 1 — Additional file 1: Title: List of priority pathogens published by the World Health Organization in 2024. Description: Table of priority pathogens by WHO. [file 12916_2026_4789_MOESM1_ESM.pdf]

Additional file 1: List of priority pathogens published by the World Health Organisation in 2024.

The list of pathogens that we will rely on, is the one that has been developed by the World Health Organization, over a 2-years consultation process. This list contains 32 priority pathogens that are likely to result in a Public Health emergency of International Concern (PHEIC).

**Family**	**Infectious Agent**	**Common Name**
Arenaviridae	*Mammarenavirus lassaense*	Lassa Fever virus
Bacteria	*Vibrio cholera (O139)*	N/A
	Yersina pestis	N/A
	Shigella dysenteriae serotype 1	N/A
	Salmonella enterica non typhoidal serovars	N/A
	Klebsiella pneumonia	N/A
Coronaviridae	*Subgenus Merbecovirus*	Middle East Respiratory Syndrome Coronavirus
	Subgenus Sarbecovirus	Severe Acute Respiratory Syndrome Coronavirus
Filoviridae	*Orthoebolavirus zairense*	Ebola virus
	Orthoebolavirus sudanens	Sudan ebolavirus
	Orthomarburgvirus marburgense	Marburg virus
Flaviviridae	*Orthoflavivirus flavi*	Yellow fever virus
	Orthoflavivirus denguei	Dengue virus
	Orthoflavivirus zikaense	Zika virus
Hantaviridae	*Orthohantavirus hantanense*	Hantaan orthohantavirus, Hantaan virus
	Orthohantavirus sinombreense	Sinnombre virus
Nairoviridae	*Orthonairovirus haemorrhagiae*	Crimean-Congo hemorrhagic fever virus (CCHF)
Orthomyxoviridae	*Alphainfluenzavirus influenzae H1, H2, H3, H5, H6, H7, H10*	Influenza A
Paramyxoviridae	*Henipavirus nipahense*	Nipah virus
Phenuiviridae	*Bandavirus dabieense*	SFTS virus
Picornaviridae	*Enterovirus coxsackielpol*	Polio virus
Poxviridae	*Orthopoxvirus variola*	Variola virus
	Orthopoxvirus monkeypox	Monkeypox virus
Retroviridae	*Lentivirus humimdef1*	Human immunodeficiency virus 1 (HIV-1)
Togaviridae	*Alphavirus chikungunya*	Chikungunya virus
	Alphavirus venezuelan	Venezuelan equine encephalitis virus

Table 1: List of priority pathogens published by the World Health Organisation in 2024.
