# Supplementary material for: Scoping review on the prioritisation of high-consequence infectious pathogens for research preparedness and response to health emergencies
Source: BMC Med. 2026 Apr 1;24:301. doi: 10.1186/s12916-026-04789-w (PMC13169742; doi:10.1186/s12916-026-04789-w)
Supplement: Supplementary file 2 — Additional file 2: Title: Inclusion and exclusion criteria. Description: Table of inclusion and exclusion criteria. [file 12916_2026_4789_MOESM2_ESM.pdf]

Additional file 2: Inclusion and exclusion criteria

This table summarises the inclusion and exclusion criteria that we used for the review of identified resources.

	**Inclusion considerations**
**Concept**	The resource must develop a **list of high-consequence infectious pathogens,** other forms of research prioritisation will be excluded (i.e. specific research domains within one disease).
**List of pathogens**	At least one of the pathogens on the list must belong to the World Health Organization list of "*priority pathogens with a high potential to cause a PHEIC [Public Health Emergency of International Concern]*" published in 2024 (Supplemental appendix 1).
**Methodology**	It is not compulsory that the methodology to establish the list is reported.
**Timeframe**	Resources published between the 1st January 2018 and the 26th of September 2024.
**Language**	No restrictions.
**Type of documents**	Only academic papers or grey literature documents will be included in the scoping review.

Table 2: List of inclusion and exclusion criteria.
