# Supplementary material for: Scoping review on the prioritisation of high-consequence infectious pathogens for research preparedness and response to health emergencies
Source: BMC Med. 2026 Apr 1;24:301. doi: 10.1186/s12916-026-04789-w (PMC13169742; doi:10.1186/s12916-026-04789-w)
Supplement: Supplementary file 3 — Additional file 3: Title: Search strategies for Overton and Google Advanced Search Tool. Description: Table with the search strategies used for the study. [file 12916_2026_4789_MOESM3_ESM.pdf]

Additional file 3: Search strategies for Overton and Google Advanced Search Tool

This table describes the search strategies used for Overton and Google Advanced Search Tool.

Overton	Google Advanced Search
(priorit* OR list*) AND (infect* OR emerg* OR pathogen* OR diseas* OR reemerg* OR outbreak* OR pandemic* OR epidemic* OR "health emergency" OR "health emergencies" OR "health crisis" or "pathogen X" OR "disease X") AND (preparedness OR readiness OR prevention OR response OR control)	**All these words**: priorities infectious diseases pathogens outbreaks pandemics epidemics<br>**Any of these words**: lists list<br>**Site or domain**: we used the domain name for each relevant organization identified.<br>**Time boundaries**: we searched from the 1st of January 2018 to the 26th of January 2024.

Table 3: Search strategies used for Overton and Google Advanced Search Tool.
