# Supplementary material for: Scoping review on the prioritisation of high-consequence infectious pathogens for research preparedness and response to health emergencies
Source: BMC Med. 2026 Apr 1;24:301. doi: 10.1186/s12916-026-04789-w (PMC13169742; doi:10.1186/s12916-026-04789-w)
Supplement: Supplementary file 4 — Additional file 4: Title: List of national and regional public health bodies. Description: Table with the national and regional public health bodies that were searched. [file 12916_2026_4789_MOESM4_ESM.pdf]

Additional file 4: List of national and regional public health bodies.

Below is the list of the institutions that were searched to identify priority lists for high-consequence infectious pathogens. This list was generated using the Pandemic PACT database to identify countries and regions with research investments in high-consequence diseases. For each WHO region, we searched total research funding amounts, and the total number of grants supported. For each region, we selected the top three funders by funding amounts or grant numbers when funding amounts were not reported. This enabled us to identify countries in each region that are likely to use priority disease lists for research funding. We then looked for national and regional public health bodies linked to the countries or the region they belonged to.

Country	Institution
South Africa	The South African Medical Research Council (SAMRC)
	National Department of Health (NDoH)
	National Institute for Communicable Diseases (NICD)
	South Africa Department of Science and Innovation
Africa	African Academy of Sciences (AAS)
	Africa CDC
United States of America	National Institutes of Health (NIH)
	Office of Disease Prevention and Health Promotion (ODPHP)
	Centers for Disease Control and Prevention (CDC)
	Bill & Melinda Gates Foundation (BMGF)
	National Science Foundation
Canada	Canadian Institutes of Health and Research
	Public Health Agency of Canada (PHAC)
India	Indian Council of Medical Research (ICMR)
	Ministry of Health and Family Welfare, Department of Health and Research
	Indo-US Science and Technology Forum
	Biotechnology Industry Research Assistance Council (BIRAC)
United Kingdom	UK Research and Innovation (UKRI)
	National Institute for Health and Care Research (NIHR)
	UK Health Security Agency (UKHSA)
	Foreign, Commonwealth & Development Office (FCDO)
Germany	Robert Koch Institute (RKI)
	Federal Ministry of Health
	German Centres for Health Research - DZIF
	The Deutsche Forschungsgemeinschaft (DFG) German Research Foundation
	German Federal Ministry of Education and Research
Europe	European Commission
	Health Emergency Preparedness and Response (HERA)
	European CDC

Pakistan	Ministry of National Health Services Regulations and Coordination, Government of Pakistan
	National Institutes of Health, Islamabad, Pakistan
	Higher Education Commission (HEC)
	The Allergy and Asthma Institute
Australia	National Health and Medical Research Council Australia
	Medical Research Future Fund
	Australian Government: Australian Research Council

Table 4: List of national and regional public health bodies searched.
