# Supplementary material for: Scoping review on the prioritisation of high-consequence infectious pathogens for research preparedness and response to health emergencies
Source: BMC Med. 2026 Apr 1;24:301. doi: 10.1186/s12916-026-04789-w (PMC13169742; doi:10.1186/s12916-026-04789-w)
Supplement: Supplementary file 5 — Additional file 5: Title: Data extraction framework. Description: Table representing the data extraction framework used for this study. [file 12916_2026_4789_MOESM5_ESM.pdf]

Additional file 5: Data extraction framework

This table shows the data items that were looked for into the included resources, this represents our data extraction framework.

**Category**	**Name of data item**	**Description**
General information	Title	As documented on the document or web page
	Author	Name of person or instituion
	Type of publication	Report, webpage, research article, comment, …
	Date published	Date published as documented on the document or web page
	Publishing institution	Name of institution(s) publishing the list
	Institution location	Indicate the location of the institution
	Institution level	Subnational, national, regional, international, global
	Reason for prioritisation undertaking	What is the purpose of the prioritisation exercise
Methods	Name of methodology	Indicate the name of the methodology if available
	Initial list	Source of initial list categorisation
	List refinement	Method for list refinement categorisation
	Criteria selection	Criteria selection categorisation
	Criteria weighting	Criteria weighting categorisation
	Pathogen scoring	Pathogen scoring categorisation
	Computing process	Computing description
	Final refinement of the list	Final list refinement categorisation
	Stakeholders	Stakeholders domain
		Stakeholders affiliation
		Overall number of stakeholders and task
		Voting stakeholders and task
	Scope of the list	Scope of the list
Results	Criteria and weightings	By criteria
	Geographical scope of the priorities	Subnational, national, regional, international, N/A
	Countries	Indicate country/countries concerned
	Regions	Indicate the region(s) concerned
Reporting, monitoring and evaluation	Planning	Objectives defined? Y/N
		Context defined? Y/N

		Resource identification (HR, financial, time) Y/N
		Review of previous studies? Y/N
	Implementation	Representativeness considered? Y/N
		Stakeholders involvement? Y/N
		How were the methods determined?
	Evaluation	Evaluation process? Y/N
		Describe evaluation plans? Y/N
		Monitoring of change? Y/N
	Publishing	Dissemination strategy? Y/N
		Implementation strategy? Y/N
		Transparency (stakeholders)? Y/N
		Transparency (methods)? Y/N
Pathogen appearances	As they appear in the resource	N/A

Table 5: Data extraction framework.
