# Supplementary material for: Scoping review on the prioritisation of high-consequence infectious pathogens for research preparedness and response to health emergencies
Source: BMC Med. 2026 Apr 1;24:301. doi: 10.1186/s12916-026-04789-w (PMC13169742; doi:10.1186/s12916-026-04789-w)
Supplement: Supplementary file 6 — Additional file 6: Title: Alignment to best practices framework. Description: WHO framework used for analysing the best practices. [file 12916_2026_4789_MOESM6_ESM.pdf]

Additional file 6: Alignment to best practices framework.

Framework for alignment to best practices (reproduced from A systematic approach for undertaking a research priority-setting exercise: guidance for WHO staff, 2020).

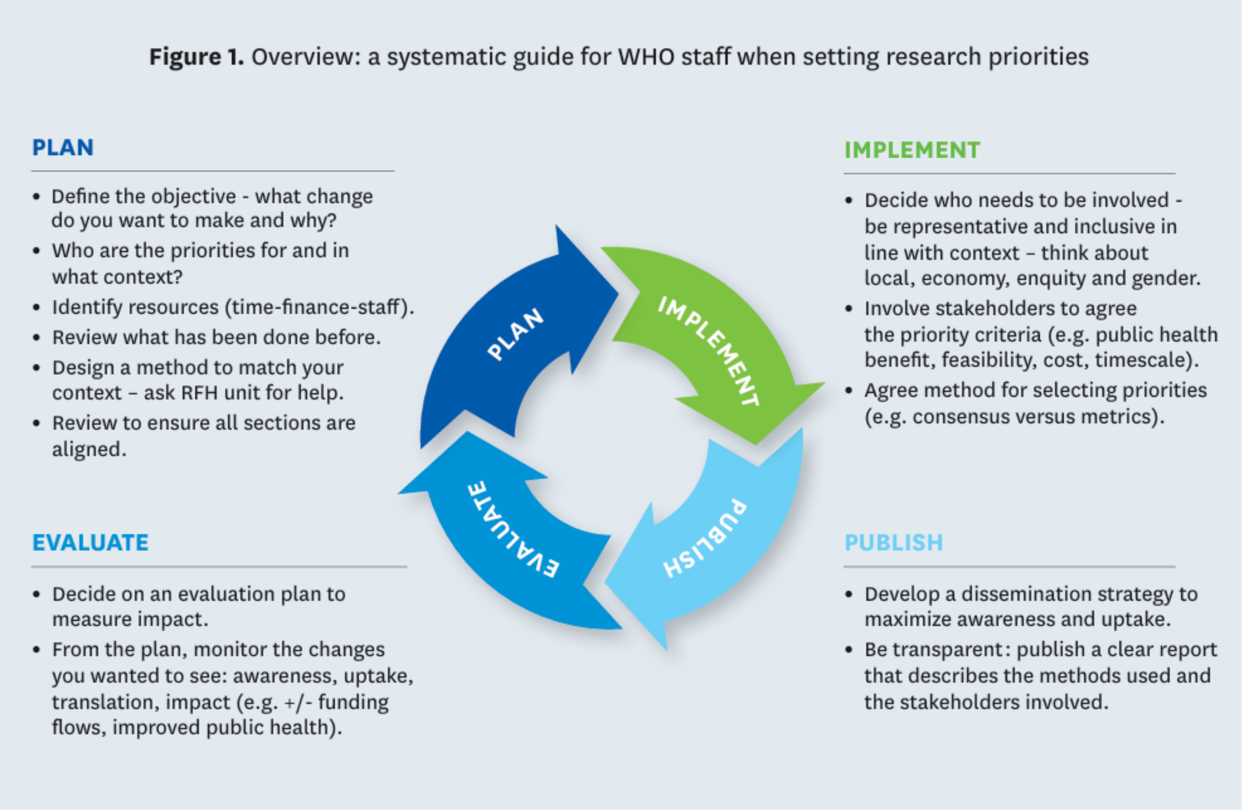

Figure S6: Framework for alignment to best practices (reproduced from A systematic approach for undertaking a research priority-setting exercise: guidance for WHO staff, 2020).
