# Supplementary figures and images for: Scoping review on the prioritisation of high-consequence infectious pathogens for research preparedness and response to health emergencies
Source: BMC Med. 2026 Apr 1;24:301. doi: 10.1186/s12916-026-04789-w (PMC13169742; doi:10.1186/s12916-026-04789-w)

**Additional file 7: PRISMA-ScR flowchart.**

Figure S7: PRISMA-ScR flowchart.

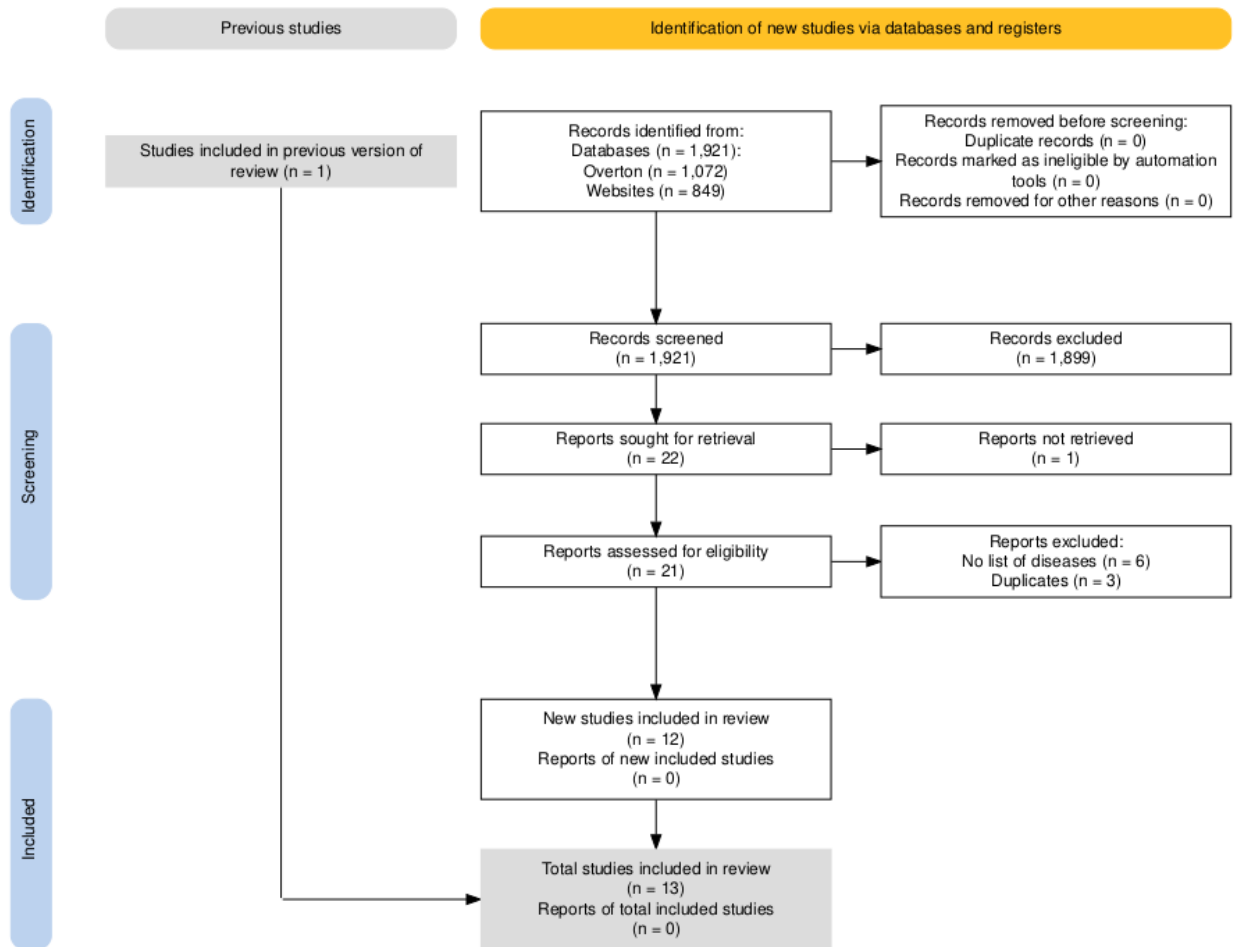

Supplement: Supplementary file 7 — Additional file 7: Title: PRISMA-ScR Flowchart. Description: Flowchart describing the process of inclusion and exclusion for this study. [file 12916_2026_4789_MOESM7_ESM.pdf]
